# The history, genome and biology of NCTC 30: a non-pandemic *Vibrio cholerae* isolate from World War One

**DOI:** 10.1098/rspb.2018.2025

**Published:** 2019-04-10

**Authors:** Matthew J. Dorman, Leanne Kane, Daryl Domman, Jake D. Turnbull, Claire Cormie, Mohammed-Abbas Fazal, David A. Goulding, Julie E. Russell, Sarah Alexander, Nicholas R. Thomson

**Affiliations:** 1 Wellcome Sanger Institute, Wellcome Genome Campus, Hinxton, Cambridgeshire CB10 1SA, UK; 2 Public Health England, 61 Colindale Avenue, London NW9 5DF, UK; 3 London School of Hygiene and Tropical Medicine, Keppel Street, Bloomsbury, London WC1E 7HT, UK

**Keywords:** *Vibrio cholerae*, long-read sequencing, antimicrobial resistance, flagella, World War One, cholera

## Abstract

The sixth global cholera pandemic lasted from 1899 to 1923. However, despite widespread fear of the disease and of its negative effects on troop morale, very few soldiers in the British Expeditionary Forces contracted cholera between 1914 and 1918. Here, we have revived and sequenced the genome of NCTC 30, a 102-year-old *Vibrio cholerae* isolate, which we believe is the oldest publicly available live *V. cholerae* strain in existence. NCTC 30 was isolated in 1916 from a British soldier convalescent in Egypt. We found that this strain does not encode cholera toxin, thought to be necessary to cause cholera, and is not part of *V. cholerae* lineages responsible for the pandemic disease. We also show that NCTC 30, which predates the introduction of penicillin-based antibiotics, harbours a functional β-lactamase antibiotic resistance gene. Our data corroborate and provide molecular explanations for previous phenotypic studies of NCTC 30 and provide a new high-quality genome sequence for historical, non-pandemic *V. cholerae*.

## 1. Introduction

*Vibrio cholerae* is the aetiological agent of cholera, a severe diarrhoeal disease that has spread globally in seven pandemics since the 1800s [1]. The sixth cholera pandemic occurred between 1899 and 1923 [2,3] and was caused by *V. cholerae* of serogroup O1 and of the classical biotype, as were the recorded pandemics prior to this [4]. The current seventh cholera pandemic began in 1961 and is caused by a different, ‘El Tor’, biotype of serogroup O1 and O139 *V. cholerae* [1]. Genome sequencing data have shown that classical *V. cholerae* form a single phylogenetic lineage, distinct from the seventh pandemic biotype El Tor (7PET) lineage which is causing the ongoing seventh cholera pandemic [3–7].

In 1931, Mitchell & Smith compiled a comprehensive analysis of medical statistics of the British Armies for World War One (WW1) [8]. These data estimated that the British Expeditionary Forces incurred 11 096 338 casualties during WW1 (equivalent casualty data for the Indian Armies were not reported) [8]. Surprisingly, despite WW1 being concurrent with the sixth cholera pandemic, the British Expeditionary Forces remained largely free of cholera throughout this period. Although cholera's epidemic potential was both recognized and feared at this time [9,10], Mitchell & Smith report that the British Expeditionary Forces experienced just 1918 cholera cases in the year 1916, 209 cases in 1917 and 450 cases in 1918. Forty-nine cholera patients died in 1917, and 106 died in 1918 [8]. All except one of these cases were associated with the Mesopotamian Expeditionary Force, which was first affected by cholera in 1916, when the disease was inadvertently transmitted from the Turkish army via a contaminated water source [8,11].

The *V. cholerae* strain ‘Martin 1’ (now dubbed NCTC 30) was the 30th bacterial culture deposited with the National Collection of Type Cultures (NCTC). It was isolated in 1916 from a British soldier convalescent in Egypt during WW1 and is believed to be of serogroup O2 (electronic supplementary material, figure S1). Because cholera was very infrequent among British troops during WW1, it is interesting that NCTC 30 was isolated at all. Moreover, the metadata describing NCTC 30 suggest that this isolate is both a unique, historical curiosity and a source of information about *V. cholerae* biology. We revived a freeze-dried culture of NCTC 30 and sequenced the genome of this isolate to completion using both long- and short-read technologies. Here, we describe a genomic and phenotypic analysis of this isolate and compare our results to previous studies of NCTC 30 biology. Given the recent 100-year anniversary of the end of WW1, it is poignant to note that our modern genomic and phenotypic data have corroborated several historical reports about the biology of NCTC 30. Taken together, these findings illustrate the rich history, as well as biological insights, that can be garnered from the study of bacterial pathogens.

## 2. Material and methods

### (a) Strains, plasmids and oligonucleotides

Bacterial strains, plasmids and oligonucleotides (Sigma-Aldrich) used in this study are listed in table 1. Strains were cultured routinely on lysogeny broth (LB) media. Plasmids were maintained in strains by culturing on LB media supplemented with 100 µg ml^−1^ ampicillin, 10 µg ml^−1^ chloramphenicol or 10 µg ml^−1^ tetracycline, where appropriate (table 1).

**Table 1.** Strains, plasmids and oligonucleotides. (Restriction enzyme recognition sites are in bold. AmpR: ampicillin resistant; CmR: chloramphenicol resistant; TcR: tetracycline resistant. AmpS: ampicillin sensitive. TcS: tetracycline sensitive.)

internal strain ID	strain name	genotype/details	source/reference
*Vibrio cholerae*			
MJD382	NCTC 30 Martin 1	isolated in 1916; Alexandria, Egypt. Non-O1/O139 (probably O2). AmpR	NCTC, batch 3
MJD439		second clone of NCTC 30. AmpR	
MJD367	NCTC 10732 CN 3534; 384/52	isolated in 1952; India. Serotype O1 Inaba, classical biotype	NCTC, batch 2
MJD389	NCTC 5395 Iraq	isolated in 1938; Iraq. Serotype O1 Ogawa, El Tor biotype. Pre-seventh pandemic. AmpS	NCTC, batch 7. Sequenced by Hu *et al.* [12]
*Escherichia coli*			
MJD839	ER2420 pACYC184	K-12 cloning strain harbouring pACYC184. CmR TcR	Francesca Short/New England Biolabs
MJD841	NEB^®^ 5-alpha	*fhuA2* Δ*(argF-lacZ)*U169* phoA glnV44* Φ*80* Δ*(lacZ)*M15* gyrA96 recA1 relA1 endA1 thi-1 hsdR17*	New England Biolabs
MJD842	NEB^®^ 5-alpha pUC19	K-12 cloning strain harbouring pUC19. AmpR	this study
MJD844	NEB^®^ 5-alpha pACYC184	K-12 cloning strain harbouring pACYC184. CmR TcR	this study
MJD847	MJD847	NEB^®^ 5-alpha harbouring pMJD61. AmpR CmR TcS	this study

plasmid name	genotype/details	source/reference	
pACYC184	low-copy cloning vector. CmR TcR	[13]	
pUC19	high-copy cloning vector, ampicillin-resistance positive control. AmpR	[14]	
pMJD61	pACYC184 *Ω*(*tet*:: *bla_CARB-like_*). AmpR CmR TcS	This study	

primer ID	other name	sequence 5'-3'	
oMJD96	BamHI_blaCARB-like-NCTC30_orf_5	CC**GGATCC**GGTTTCAGTGCCTAATGCTTTAAGTTAAGATG	
oMJD97	blaCARB-like-NCTC30_orf_SalI_3	CC**GTCGAC**ATCAACGCGACTGTGATGTATAAACTTCAA	
oMJD88	blaCARB-like-NCTC30_int_5	TGGGGTCACATACATGAAGTCT	
oMJD89	blaCARB-like-NCTC30_int_3	CAGCAATACTCCACTTCACTG	
oMJD98	pACYC184_tet_seq_Pf	GTTAAATTGCTAACGCAGTC	
oMJD99	pACYC184_tet_seq_Pr	GTGAATCCGTTAGCGAGGTG	
oMJD135	VC_2135_check_Pf	GTCAGGCAGATAGCTCAAACT	
oMJD136	VC_2135_check_Pr	CTCATTGCTACCTCTGATGCC	

### (b) Bacterial rehydration and recovery

Lyophilized *V. cholerae* cultures were recovered according to the method published by Public Health England Culture Collections (https://www.phe-culturecollections.org.uk/). For full details, see the electronic supplementary material, Methods. Briefly, lyophilized bacterial stocks were rehydrated and cultured on LB media overnight (passage 1). Colonies were purified on LB and thiosulfate-citrate-bile salts-sucrose (TCBS) agar, a medium selective for *Vibrio* species (passage 2). Colonies from TCBS plates (or from LB plates if growth on TCBS agar was poor) were cultured in LB liquid media for 24 h at 37°C (passage 3). Glycerol stocks from these cultures were stored at −80°C.

### (c) Genomic DNA isolation and sequencing

Total nucleic acids were extracted for sequencing from *V. cholerae* using the Masterpure Complete DNA and RNA Purification kit (Epicentre, no. MC85200), with modifications to the manufacturer's instructions. DNA was isolated from two independent clones of NCTC 30 picked at passage 2 (dubbed MJD382 and MJD439) and one clone of NCTC 5395 (MJD389), a strain that is closely related to 7PET *V. cholerae* [12]. All clones had been frozen at passage 3. Single colonies isolated from these frozen stocks (passage 4) were used to lawn LB agar plates, which were incubated overnight at 37°C (passage 5) and used for genomic DNA (gDNA) isolation. Full details are provided in the electronic supplementary material, Methods.

gDNA from NCTC 30 batch 3 was sequenced using the Illumina X10 and the PacBio RSII platforms at the Wellcome Sanger Institute. DNA fragments of approximately 450 bp were produced from 0.5 µg gDNA for Illumina library creation and were sequenced on a 150 bp paired-end run. Approximately 10 µg gDNA was used for PacBio sequencing, using polymerase version P6 and C4 sequencing chemistry reagents. gDNA from NCTC 30 batch 4 was sequenced on the PacBio Sequel platform.

### (d) Genome assembly and annotation

Single-contig assemblies were generated for each of the two NCTC 30 chromosomes from PacBio read data, using HGAP v3 and the RS_HGAP_Assembly.2 protocol via SMRT Portal running SMRT Analysis v2.3.0.140936.p5.167094 [15]. These sequences were circularized using Circlator v1.5.3 [16] using the assembly and the corrected reads. A final assembly was obtained by using the circularized sequences as a reference for re-assembly of the PacBio reads with the RS_Resequencing.1 protocol, which was corrected using Quiver v1. Assemblies were annotated using Prokka v1.5 [17] and a genus-specific database [18]. The PacBio sequencing reads covered the finished assembly to an average depth of 148.01 X. For parameter details, see the electronic supplementary material, Methods.

Short-read data used for pangenome analyses (electronic supplementary material, table S1) were assembled using SPAdes v3.8.2 [19] as part of a high-throughput analysis pipeline and annotated using Prokka v1.5 [17,20]. Sequences that were available only as assemblies (i.e. for which the raw sequencing reads were not available in reference databases for *de novo* assembly) were similarly annotated using Prokka v1.5 for uniformity within the dataset.

### (e) Genome visualization, synteny plots and antimicrobial resistance gene detection

The NCTC 30 genome was visualized using the GView web server (https://server.gview.ca/), which relies on CGView [21]. Synteny plots were produced using Easyfig [22] and ACT [23], which rely on BLASTn [24] for sequence comparisons. A minimum identity percentage of 85%, maximum *e*-value of 0.001 and minimum length of 0 were chosen as BLASTn cut-offs for Easyfig visualization purposes. Antimicrobial resistance genes were detected in the genome assembly using the ResFinder web server v3.1.0 [25] with default settings (90% identity, 60% minimum length) and database version 2018-02-19.

### (f) Phylogenetic analysis and lineage assignment

A pangenome was constructed from annotated genome assemblies of 198 *V. cholerae* isolates and three *Vibrio* spp. using Roary v1.007001 [26], with options: ‘-e --mafft -s -cd 97’. A core-gene alignment of 2622 genes was produced. This alignment was trimmed using trimAl v1.2 [27], and non-variable positions were removed using SNP-Sites v2.3.2 [28]. A maximum-likelihood phylogenetic tree was constructed from this alignment of 192 451 variant sites using IQ-Tree v1.5.5 [29], under the general time reversible (GTR) and ascertainment bias correction (ASC) models, the latter of which is optimized for accepting alignments that consist entirely of variable nucleotides [30]. Five thousand ultrafast bootstrap approximations [31] and approximate likelihood ratio tests [32] were performed. Phylogenetic trees were visualized using FigTree v1.4.3 (http://tree.bio.ed.ac.uk/software/figtree/) and iTOL [33] and were annotated manually.

*Vibrio cholerae* genomes were assigned to phylogenetic lineages based on previous reports [7], their position in the maximum-likelihood phylogeny and with the support of a hierarchical Bayesian analysis of population structure (BAPS) [34]. Private single nucleotide polymorphisms (SNPs) (i.e. SNPs found in one genome only) were removed from the variable nucleotide alignment used for phylogenetic analysis using extract_PI_SNPs.py (https://gist.github.com/jasonsahl/9306cd014b63cae12154), to produce an alignment of 136 993 parsimony-informative variable nucleotides, used as the input for BAPS (with options *L* = 3, *K* = 500).

### (g) Plasmid extraction, polymerase chain reaction and molecular cloning

Plasmids were isolated from *Escherichia coli* using the QIAprep Spin Miniprep kit (Qiagen, no. 27104). Reaction intermediates were purified using the QIAquick polymerase chain reaction (PCR) Purification kit (Qiagen, no. 28104). Full details of the *bla_CARB-like_* cloning protocol are reported in the electronic supplementary material, Methods—briefly, *bla_CARB-like_* was amplified from MJD382 gDNA using oMJD96 and oMJD97 and Phusion^®^ high-fidelity DNA polymerase (NEB, no. M0530S). This insert and pACYC184 were digested with BamHI and SalI (NEB, no. R3136S and no. R3138S), and pACYC184 was treated with rSAP (NEB, no. M0371S). Digested insert and vector were purified, mixed in a molar ratio of approximately 3 : 1 and ligated using T4 DNA ligase (NEB, no. M0202S). Competent *E. coli* was transformed with ligation mixtures as per the manufacturer's instructions. Constructs were verified by PCR using oMJD88 and oMJD89, and by Sanger sequencing (GATC/Eurofins) with oMJD98 and oMJD99.

### (h) Confirmation of genomic observations

The Illumina short-reads for NCTC 30 were mapped to the NCTC 30 assembly using SMALT v0.5.8 (http://www.sanger.ac.uk/science/tools/smalt-0), and visualized using Artemis and BamView [35,36] (electronic supplementary material, figure S3). The *flrC* mutation was confirmed by amplifying *flrC* from *V. cholerae* gDNA using Phusion^®^ and primers oMJD135 and oMJD136. The resultant amplicon was purified and sequenced (GATC/Eurofins).

### (i) Growth curves

In order to assess bacterial growth kinetics, single colonies of *V. cholerae* were suspended in 0.5 ml LB broth by vortexing (10 s). Two microlitres of this suspension were used to inoculate 150 µl LB in a 96-well microtitre plate (Corning CoStar no. 3595, flat-bottomed). A gas-permeable seal was applied to the plate, which was incubated at 37°C with shaking in a BMG Fluostar Omega microtitre plate reader for 24 h. Details of the incubation program are reported in the electronic supplementary material, Methods.

### (j) Antibiotic sensitivity assay

Ampicillin sensitivity was assessed using MICEvaluator Ampicillin test strips (Oxoid, no. MA0110F). Lawns of bacterial growth were prepared as for gDNA isolations, and plasmid-harbouring strains were cultured with the selection. Sections of the lawn were suspended in 1.0 ml LB medium. The OD_600_ of this suspension was normalized to 0.5, and cotton swabs were used to inoculate LB agar with these standardized suspensions. Plates were dried for 15 min, before an MICEvaluator test strip was applied to the plate surface. Plates were incubated for 20 h at 37°C. Break points were determined using the manufacturer's instructions.

### (k) Motility assay

In order to determine the motility of *V. cholerae* strains, bacterial colonies were picked and suspended in 0.5 ml LB media. Two microlitres of this suspension were used to inoculate motility LB agar plates (0.3% agar in 140 mm dishes). The pipette tip was pushed through the agar surface during inoculation. Plates were incubated face up at 37°C.

### (l) Transmission electron microscopy

Bacterial morphologies were determined using transmission electron microscopy. Bacterial colonies were picked and suspended in 0.5 ml sterile water. The suspension (4 µl) was applied to a glow-discharged Formvar carbon film copper transmission electron microscopy grid (FCF-100-Cu) and mixed with ammonium molybdate solution (2.5% final concentration). Images were acquired using an FEI Tecnai G2 Spirit BioTWIN.

## 3. Results and discussion

### (a) Sequencing and analysis of the NCTC 30 genome

Previously published data indicated that NCTC 30 was not of serogroup O1 and was therefore unlikely to be a sixth pandemic *V. cholerae* isolate [37] (the isolate is likely to be of serogroup O2; electronic supplementary material, figure S1). We were intrigued by this, since it was isolated from a hospitalized patient reportedly suffering from diarrhoea [37]. We revived NCTC 30 from batch 3 of NCTC's freeze-dried stocks, a lyophilized bacterial culture that was prepared in 1962 (electronic supplementary material, figure S1). Given the age of this isolate, we used long- and short-read technologies to sequence high-molecular weight gDNA from a minimally passaged culture of NCTC 30 to avoid sequencing a spontaneous mutant.

**Figure 1.**
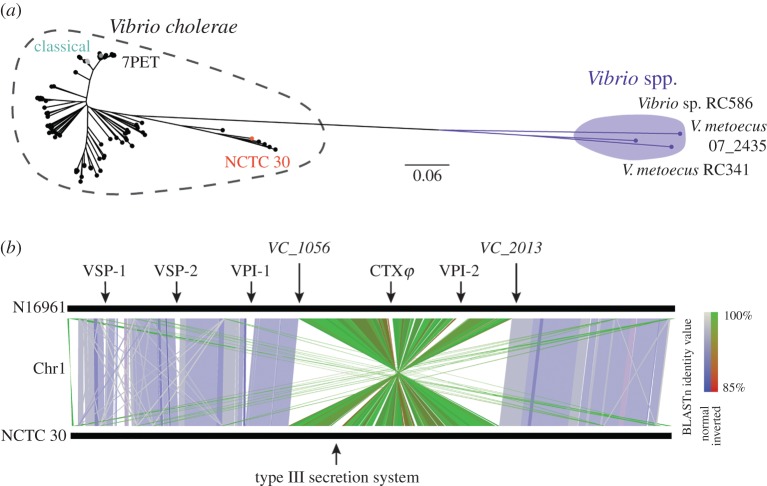
The NCTC 30 genome sequence and its relatedness to *Vibrio cholerae*. (*a*) An unrooted maximum-likelihood phylogeny shows that NCTC 30 clusters together with six isolates that have been previously reported to be *Vibrio cholerae* (electronic supplementary material, table S1). Pandemic lineages are highlighted. Scale bar denotes the number of mutations *per* variable site. (*b*) An inversion of approximately 1 040 746 bases between *VC_1056* and *VC_2013* was identified in NCTC 30 chromosome 1, relative to that of the N16961 reference sequence. NCTC 30 lacks the pathogenicity islands found in 7PET or classical *V. cholerae*. The NCTC 30 sequence has been reversed for illustrative purposes.

We constructed a pangenome using a collection of 197 other publicly available *V. cholerae* genome sequences, and those of three *Vibrio* spp. that are closely related to *V. cholerae*. A maximum-likelihood phylogeny produced from the resultant core-gene alignment of 2622 genes showed that NCTC 30 is more closely related to *Vibrio cholerae* sequences than to other members of the *Vibrio* genus, although NCTC 30 is part of a clade that is separated from many of the *V. cholerae* in this collection (figure 1*a*; electronic supplementary material, table S1). This observation is logical when considered together with a taxonomic study of *V. cholerae* performed in 1970, which questioned whether NCTC 30 is a true member of the *V. cholerae* species [38]. The phylogenetic separation which we observed is likely to reflect the phenotypic and molecular differences that questioned the classification of NCTC 30 [38]. However, our data do indicate that NCTC 30 is a *V. cholerae* isolate, as are its closest relatives (electronic supplementary material, table S1; [7]).

The NCTC 30 genome assembly comprised two circularized contigs, one corresponding to the larger chromosome 1 of 2 922 904 bases, and one to the smaller chromosome 2 of 1 029 451 bases (electronic supplementary material, figure S2). A comparison between these sequences and those of the O1 El Tor *V. cholerae* reference strain, N16961 [39] revealed a large inversion in NCTC 30 chromosome 1 of approximately 1 040 746 bases, between genes *VC_1056* and *VC_2013* (figure 1*b*). The inversion does not encompass the *crtS* locus and should not interfere with the rate and timing of chromosome 2 replication [40]. We confirmed that this inversion was not an artefact of genome assembly by mapping the NCTC 30 short-reads to the PacBio assembly and to N16961, identifying paired-end reads that mapped to either side of the inversion junction, as well as individual reads whose sequence spanned the junction itself (electronic supplementary material, figure S3). This was confirmed further using sequencing data from a second gDNA isolation from MJD382, as well as from MJD439, an independent colony of NCTC 30 separated from MJD382 at passage 1 (see Material and methods; electronic supplementary material, table S2).

### (b) NCTC 30 does not produce flagella

**Figure 2.**
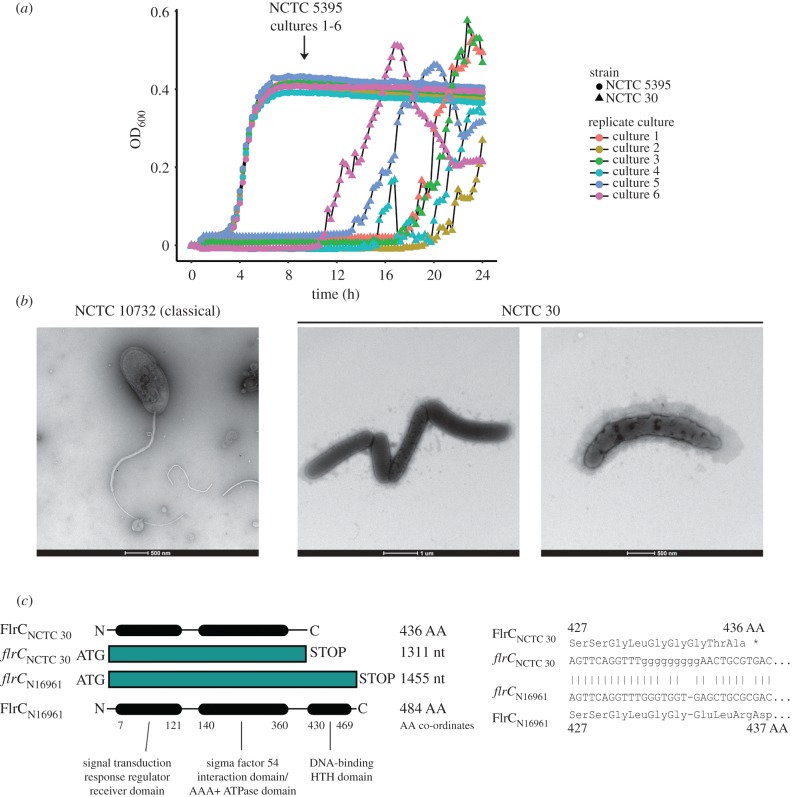
NCTC 30 is impaired in its ability to produce flagella. (*a*) NCTC 30 has a growth defect at 37°C relative to NCTC 5395. Under these conditions, *V. cholerae* does not grow to an OD_600_ exceeding 1.0—accordingly, a non-logarithmic *Y*-axis scale has been used. Representative data from single biological experiments are reported, figure produced using R v3.3.2 and ggplot2 [41]. (*b*) Transmission electron microscopy demonstrates that NCTC 30 does not produce the polar monotrichous flagellum that is characteristic of *V. cholerae*, represented here by NCTC 10732, a classical biotype strain. (*c*) NCTC 30 contains a frameshift mutation in the 3′-end of *flrC* relative to the N16961 reference sequence, predicted to produce a truncated polypeptide lacking the C-terminal FlrC DNA binding domain. FlrC domains were annotated using InterProScan (https://www.ebi.ac.uk/interpro) [42]. *flrC* 3′ sequences were aligned using BLASTn [24]. *flrC* open reading frame: grey box. FlrC protein domains: black ovals. Figures not to scale.

We found NCTC 30 to be extremely difficult to culture under our standard laboratory conditions—it has a growth defect on rich media at 37°C relative to other *V. cholerae* in our collection. Exemplar growth kinetic data from liquid culture illustrate this (figure 2*a*). An examination by electron microscopy showed that NCTC 30 lacked monotrichous flagella, in contrast with the phenotype expected for *V. cholerae* (figure 2*b*), and we confirmed that NCTC 30 is not motile (electronic supplementary material, figure S4). Note that we used NCTC 10732 as a control strain for electron microscopy experiments, because the majority of flagella studies in this species have been performed using classical *V. cholerae* [43–45].

The genes and proteins involved in *V. cholerae* flagellum expression are well-characterized [43–45]. We hypothesized that disruption to this pathway might have caused the observed phenotypes (figure 2*a*,*b*; electronic supplementary material, figure S4). We identified a frameshift in the 3′ region of *flrC* (*VC_2135*) in NCTC 30, which encodes the FlrC response regulator governing the expression of Class III flagellum biosynthesis genes [45]. Class III genes encode the flagellar cap, the MotX motor component and the core flagellin FlaA [43]. All Class III genes were intact in NCTC 30. The frameshift was predicted to truncate FlrC, removing the last 48 amino acids from the C-terminus of the protein (figure 2*c*). This region is predicted to serve as the DNA binding domain of the response regulator; accordingly, we believe that this frameshift prevents FlrC *trans*-activating the Class III flagellum biosynthesis genes in NCTC 30, abolishing its ability to manufacture flagella. The morphology of NCTC 30 is consistent with that of an *flrB* targeted mutant [45]. FlrB acts as the sensor kinase in the FlrBC two-component system, and because both proteins cooperate to regulate Class III gene expression, this would explain why *flrB* and *flrC* mutations appear to phenocopy one another.

In contrast with our observations, Davis & Park reported that NCTC 30 expressed monotrichous flagella [46]. This report was submitted for publication in April 1962 [46], prior to the preparation of batch 3 of NCTC 30 (electronic supplementary material, figure S1). We hypothesized that the *flrC* mutation may have arisen during the preparation of batch 3, during long-term storage [47], or during passage in our laboratory. We confirmed that this mutation was present in the genome sequences of MJD382 and MJD439, and used the high-accuracy Illumina short-read data to verify that the repetitive sequence was not an artefact of long-read assembly. This suggested either that this mutation predated the introduction of the strain into our laboratory or had arisen immediately upon rehydration of our lyophilized stock. Therefore, we prepared gDNA from batch 4 of NCTC 30 in a laboratory separate to that in which MJD382 and MJD439 were handled. Batch 4 was lyophilized in 1985 from a culture of batch 3 bacteria (electronic supplementary material, figure S1). We amplified and sequenced *flrC* from this preparation and confirmed that the *flrC* frameshift mutation was present in batch 4 of NCTC 30 (electronic supplementary material, figure S5). This indicates strongly that the mutation arose either during or prior to the preparation of batch 3 of this lyophilized culture and that this mutation ought to be present in NCTC 30 cultures which are purchased from NCTC in the future.

### (c) Virulence determinants harboured by NCTC 30

In the absence of any clinical data, we explored the genome of NCTC 30 to determine if it was likely to be the aetiological agent of ‘choleraic diarrhoea’ [37]. CTX*φ*, the lysogenic bacteriophage that encodes the cholera toxin (CT), was absent in its entirety from both chromosomes of NCTC 30 (figure 1*b*; electronic supplementary material, figure S6). Several other pathogenicity islands have been associated with virulence in *V. cholerae* [5,48,49], and we used synteny comparisons and the mapping of NCTC 30 reads to the N16961 reference to confirm that NCTC 30 lacks *Vibrio* pathogenicity islands 1 and 2 (VPI-1 and VPI-2), *Vibrio* seventh pandemic islands 1 and 2 (VSP-1, VSP-2) and the integrative conjugative element SXT/R391 (figure 1; electronic supplementary material, figure S6 and table S3).

As NCTC 30 lacked CTX*φ*, we hypothesized that an alternative virulence factor may have rendered this strain pathogenic. Even in the absence of CT, *V. cholerae* can express secondary virulence factors including a haemolysin, the MARTX toxin, a mannose-sensitive haemagglutinin type IV pilus (MSHA), a heat-stable enterotoxin and a type III secretion system (T3SS) [1,50–53]. Non-O1/O139 *V. cholerae* lacking CT can cause various forms of diarrhoea, some using T3SS to achieve this [50,54,55]. Otherwise-uncharacterized cytotoxic factors can lead to non-O1/O139 *V. cholerae* causing non-diarrhoeal infections such as sepsis [56].

**Figure 3.**
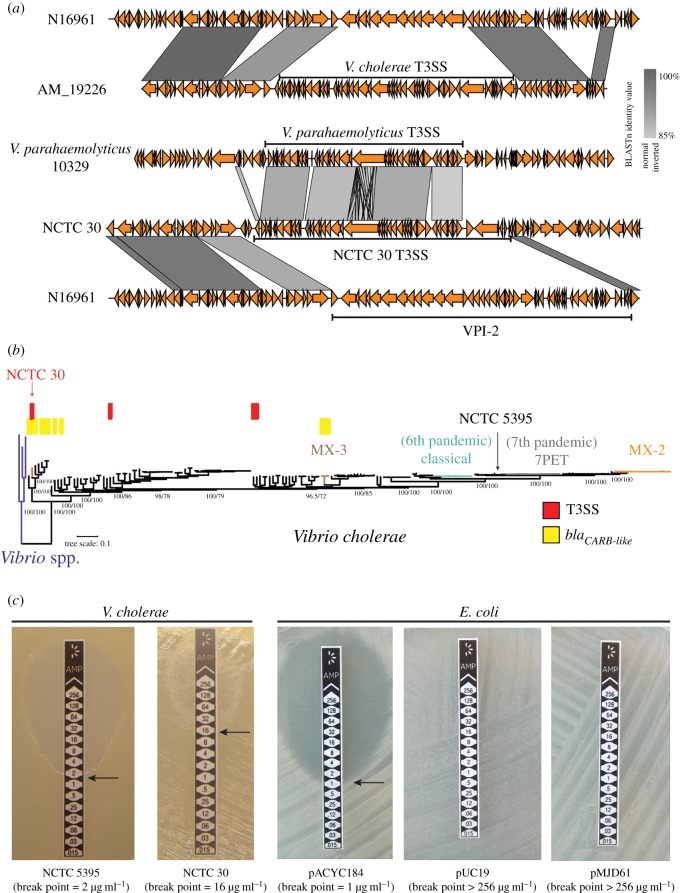
NCTC 30 is resistant to β-lactams and harbours virulence genes similar to those of *V. parahaemolyticus*. (*a*) The T3SS encoded by NCTC 30 is most similar to one encoded by *V. parahaemolytius* strain 10329 and is dissimilar to that encoded by *V. cholerae* AM_19226 [50]. The chromosomal integration locus for T3SS in both NCTC 30 and AM_19226 is the same. The genes flanking the T3SS in *V. parahaemolyticus* are not similar to those of *V. cholerae*. (*b*) The phylogenetic tree from figure 1*a* is presented, rooted on the *Vibrio* spp. outgroup. Select *V. cholerae* lineages [7] are indicated. Genomes that contain homologues of the T3SS and β-lactamase genes found in NCTC 30 (95% amino acid identity cut-off) are indicated. NCTC 30 is the only isolate in the collection in which these elements are coincident. Approximate likelihood ratio test result and bootstrap support percentages for major nodes are shown. Scale bar denotes the number of mutations *per* variable site. (*c*) NCTC 30 resists ampicillin to a greater extent than NCTC 5395. Break points are indicated with arrows. The faint growth of NCTC 30 close to the test strip above the 16 µg ml^−1^ position resembles satellite colonies that emerge owing to β-lactam degradation by enzyme secreted by adjacent bacterial culture. pMJD61, containing *bla_CARB-like_*, confers ampicillin resistance to the same level as the pUC19 ampicillin-resistance plasmid in *E. coli*.

We examined the NCTC 30 genome for the presence of the *zot*, *ace*, *hlyA*, *rtxA*, *rtxC*, *hapA*, MSHA and heat-stable enterotoxin accessory virulence genes (electronic supplementary material, table S3), and identified a genomic island in NCTC 30 which encodes a putative T3SS. This island is integrated between *VC_1757* and *VC_1810*, in place of VPI-2 in N16961 (figure 1*b*). This T3SS is more similar to the T3SS found in the genome of *Vibrio parahaemolyticus* strain 10329 [57] than the T3SS found in *V. cholerae* AM_19226, the strain used to characterize T3SS activity in *V. cholerae* [50,51] (figure 3*a*). A handwritten note on the NCTC's internal quality check card for NCTC 30 refers to ‘intermediate *V. cholerae*/*V. parahaemolyticus*' (electronic supplementary material, figure S1). No further information is available to explain why this note was made, though the presence of the genes encoding a *V. parahaemolyticus* T3SS in this isolate is intriguing.

Three *V. cholerae* genomes in our dataset, TUC_T2734, 1587 and 623-39, lack CTX*φ* but contained genes similar to those of the NCTC 30 T3SS (figure 3*b*; BLASTp similarity cut-off of 95%). Isolates 1587 and 623-39 have previously been reported to encode T3SS [58]. It may be that the T3SS encoded by these isolates, and NCTC 30, was responsible for clinical symptoms that led to the isolation of these bacteria. We also cannot exclude the possibility that the patient was co-infected with another pathogen in addition to NCTC 30, either an O1 *V. cholerae* or another bacterium such as enterotoxigenic *E. coli* [59,60], which might also have caused ‘choleraic diarrhoea’.

### (d) NCTC 30 displays reduced susceptibility to ampicillin

Davis & Park reported that NCTC 30 was resistant to penicillin, at a concentration which partially inhibited the growth of NCTC 5395 [46]. ResFinder [25] identified one resistance gene in the NCTC 30 genome that is 99.77% identical in nucleotide sequence (two base mismatches) to the *bla_CARB-7_* gene, GenBank accession no. AF409092. This *bla_CARB_*-like variant β-lactamase gene, dubbed *bla_CARB-like_*, is located within the super-integron of NCTC 30 chromosome 2 (electronic supplementary material, figure S2). Although the super-integron is a highly repetitive region of the genome [61], we were able to assemble this region fully using our long-read data.

The presence of a DNA sequence encoding a putative β-lactamase neither means that the gene is itself expressed, nor that the gene function is that which it has been predicted to be. We used MICEvaluator strips to test semi-quantitatively whether NCTC 30 was resistant to ampicillin. Consistent with previous reports, we found that NCTC 30 has decreased sensitivity to ampicillin relative to NCTC 5395, the strain to which NCTC 30 had been compared previously [46] (MICEvaluator break points of 16 versus 2 µg ml^−1^; figure 3*c*). We cloned *bla_CARB-like_* into pACYC184, a low-copy vector that confers resistance to chloramphenicol and tetracycline [13] (electronic supplementary material, figure S7). The resultant plasmid, pMJD61, rendered *E. coli* resistant to ampicillin to a level equivalent to that conferred by pUC19, a plasmid encoding a β-lactamase [14] (figure 3*c*). We conclude that *bla_CARB-like_* encodes a functional ampicillin-resistance determinant, which can be expressed in members of the Vibrionaceae and the Enterobacteriaceae.

We used BLASTx to scan the nr database using the translated *bla_CARB-like_* sequence as a query. The most similar sequences (99% amino acid similarity) were those of the *V. cholerae* β-lactamases CARB-7 and CARB-9. CARB-7 was first described in an environmental *V. cholerae* isolated in Argentina that resisted ampicillin to an minimum inhibitory concentration (MIC) of 256 µg ml^−1^ [62]. Like *bla_CARB-like_*, the gene encoding CARB-7 is located within the super-integron of chromosome 2 [62]. CARB-9 is also an integron-encoded β-lactamase, first identified in environmental non-O1/O139 *V. cholerae* from Argentina [63]. The isolate that harboured CARB-9 resisted ampicillin to an MIC of 64 µg ml^−1^ [63].

Ten *bla_CARB-like_* homologues were present in our pangenome dataset (BLASTp similarity cut-off of 95%), in strains closely related to NCTC 30 as well as in the MX-3 lineage of O1 *V. cholerae*, isolated in Mexico during 2000 [7] (figure 3*b*; electronic supplementary material, table S1). Although a β-lactamase gene, *bla_CARB-2_*, was reported by Domman *et al*. to be present in MX-3, the phenotypic data available for strain 82711, also containing *bla_CARB-2_*, indicated that this strain was not resistant to penicillin-derived antimicrobials [7]. We suggest that this apparent discordance may reflect variety in β-lactam resistance phenotypes in *V. cholerae*; *bla_CARB-2_* might elevate β-lactam resistance, but not to a level sufficient to classify a strain as ‘resistant’ to an antimicrobial.

NCTC 30 predates the introduction of penicillin as an antibiotic, the antimicrobial activity of which was first reported by Fleming in 1929 [64]. Consequently, NCTC 30 is unlikely to have acquired its drug resistance phenotype in response to selective pressures imposed by the therapeutic use of antibiotics. It is also worth noting that β-lactams are not recommended for the treatment of cholera [65,66]. We suggest that NCTC 30 may possess *bla_CARB-like_* in order to protect itself from antibiotics in its environment—i.e. to defend itself against antibiotic-producing microorganisms with which it might coexist in the environment. This may explain why this strain, although resistant to ampicillin to a greater extent than other *V. cholerae*, does not resist the antibiotic completely; it may be that *bla_CARB-like_* is expressed at levels sufficient to protect NCTC 30 from diffuse, low-concentration antibiotics present in an environment.

## 4. Conclusion

Piecing together the history of cholera pandemics requires not only an understanding of pandemic *V. cholerae* lineages but also a view of the more diverse non-pandemic *V. cholerae* that are contemporaneous with the pandemics. NCTC 30 was isolated at a time when the sixth cholera pandemic was waning [2,3]. Very few *V. cholerae* isolates and genome sequences are available from this time period, making NCTC 30 a valuable isolate for future evolutionary studies of the *V. cholerae* species.

We have presented a genomic and phenotypic characterization of this non-pandemic, 102-year-old isolate, and have compared it to other *V. cholerae*, including strains to which it has been compared directly in previous reports [46]. The unusual phylogenetic position of NCTC 30 suggests that this sequence has considerable use in the study of the non-O1/O139 and non-pandemic *V. cholerae*, and by providing the genome sequence of NCTC 30 as a community resource, we complement the availability of live NCTC 30 as a biological resource for researchers. Although this isolate proved difficult to manipulate experimentally, we have been able to explore three key historical observations made about this strain: a molecular explanation for its decreased sensitivity to β-lactams relative to NCTC 5395, phylogenetic data on its relationship to the *V. cholerae* species and evidence for a pathogenicity island that may have been responsible for causing diarrhoea in 1916. We have also described differences between our stocks of NCTC 30 and previous reports—namely, the ability of NCTC 30 to produce flagella. Given the age of this isolate, these differences might be owing to genetic changes that occurred during its long-term storage.

We have demonstrated that *bla_CARB-like_* is a functional ampicillin-resistance gene when introduced into an *E. coli* cloning strain. Genomic and phenotypic characterization of NCTC 1, a *Shigella flexneri* isolated during WW1, showed that this strain was also resistant to penicillin (among other antimicrobials) despite predating the antibiotic era [67]. The fact that *bla_CARB-like_* is located within the *V. cholerae* super-integron suggests that this gene may have been acquired horizontally, and its compatibility with another bacterial genus is intriguing. These data re-iterate the fact that the presence of antimicrobial resistance genes in bacterial pathogens predates the introduction of antibiotic therapies [68,69].

## Supplementary Material

Extended methods, supplementary figures and references

## Supplementary Material

Table S1

## Supplementary Material

Table S2

## Supplementary Material

Additional materials

## Data accessibility

Next-generation sequencing data generated in this study have been deposited into the European Nucleotide Archive (ENA; http://www.ebi.ac.uk/ena) under accession number ERP110583. The NCTC 30 genome assembly has been deposited into the ENA under accession numbers LS997867 and LS997868. All other data, including raw amplicon sequencing reads, sequence alignments and growth curve data points, are available as part of the electronic supplementary material.
