# Supplementary material for: The history, genome and biology of NCTC 30: a non-pandemic Vibrio cholerae isolate from World War One
Source: Proc Biol Sci. 2019 Apr 10;286(1900):20182025. doi: 10.1098/rspb.2018.2025 (PMC6501683; doi:10.1098/rspb.2018.2025)
Supplement: Extended methods, supplementary figures and references [file rspb20182025supp1.pdf]

Supplementary material for

The history, genome, and biology of NCTC 30, a non-pandemic *Vibrio cholerae* isolate from World War One.

Matthew J. Dorman, Leanne Kane, Daryl Domman, Jake D. Turnbull, Claire Cormie, Mohammed-Abbas Fazal, David A. Goulding, Julie E. Russell, Sarah Alexander & Nicholas R. Thomson

These supplementary materials include:

Supplementary Methods.

Table S1. Additional genomes used in this study (attached .xls spreadsheet).

Table S2. Accession numbers for NCTC 30 genome sequencing (attached .xls spreadsheet).

Table S3. Presence of select accessory virulence factor genes in the NCTC 30 genome.

Figures S1-S7.

Supplementary references for genomes used in this study.

Additional materials that support this study are available in Figshare:

https://doi.org/10.6084/m9.figshare.c.4450613

Supplementary Methods (cited references are listed in the main text)

Bacterial rehydration and recovery

NCTC 30, NCTC 5395 and NCTC 10732 were recovered from lyophilised stocks according to the method published by Public Health England Culture Collections (https://www.phe-culturecollections.org.uk/). Ampoules containing lyophilised bacterial stocks were broken under sterile conditions, and the contents were rehydrated using 0.5 ml LB medium for five minutes at room temperature. This suspension was mixed well and applied to LB agar plates, which were incubated overnight at 37 ˚C (passage 1). Three well-isolated colonies from these plates were single-colony purified onto both LB and TCBS agar, a medium selective for *Vibrio* species (passage 2). Colonies were taken from TCBS plates (or from LB plates if growth on TCBS agar was poor) and used to inoculate 3 ml LB liquid media, which was incubated for 24 h with shaking (180 rpm) at 37 ˚C (passage 3). Cultures were mixed with glycerol (25% v/v final concentration) and frozen at -80 ˚C.

Genomic DNA isolation

Total nucleic acids were extracted from *V. cholerae* using the Masterpure Complete DNA and RNA Purification kit (Epicentre, #MC85200), with modifications to the manufacturer's instructions. DNA was isolated from two independent clones of NCTC 30 picked at passage 2 (dubbed MJD382 and MJD439) and one clone of NCTC 5395 (MJD389), a strain that is closely related to 7PET *V. cholerae* [12]. All clones had been frozen at passage 3. Single colonies isolated from frozen bacterial stocks (passage 4) were used to lawn LB agar plates, which were incubated overnight at 37 ˚C (passage 5). Five loopfuls of bacterial lawn were added to 300 µl Tissue & Cell Lysis Solution supplemented with Proteinase K. Samples were vortexed (10 sec), and incubated at 65 ˚C with intermittent vortexing for 20-25 min or until the suspension had cleared. Samples were treated with RNAse A for 30 min, and chilled on

ice. Proteins were precipitated using MPC Protein Precipitation Solution (150 µl), followed by centrifugation (16,000 x *g*; 10 min; 4 ˚C). Residual protein was precipitated by re-treating the samples with MPC reagent (30 µl). Genomic DNA (gDNA) was precipitated from the cleared supernatant using room-temperature isopropanol, collected by centrifugation (16,000 x *g*; 10 min; 4 ˚C), washed twice with 1 ml room-temperature 70% v/v ethanol, dried, and resuspended in 80 µl nuclease-free water. EDTA was excluded from the resuspension solution, to avoid interference with PacBio sequencing chemistry.

NCTC 30 genome assembly, annotation, and quality checks

Single-contig assemblies were generated for each of the two NCTC 30 chromosomes from PacBio read data, using HGAP v3 and the RS_HGAP_Assembly.2 protocol *via* SMRT Portal running SMRT Analysis v2.3.0.140936.p5.167094 [15]. Subreads were filtered for a minimum length of 500 bases; minimum polymerase read quality and length were set to 0.8 and 100, respectively. For assembly, the minimum seed read length was set to 6,000, and the following options were passed to BLASR: '-noSplitSubreads -minReadLength 200 -maxScore -1000 -maxLCPLength 16'. The expected genome size was set to 5 Mbp with a target coverage of 30. The resultant assembly comprised two contigs, one *per V. cholerae* chromosome. These sequences were circularised using Circlator v1.5.3 [16] using the assembly and the corrected reads. A final assembly was obtained by using the circularised sequences as a reference for re-assembly of the PacBio reads with the RS_Resequencing.1 protocol (minimum subread length of 50 bases, minimum polymerase read quality of 75%, minimum polymerase read length of 50 bases, BLASR maximum divergence of 30% and minimum anchor size of 12), which was corrected using Quiver v1. Assemblies were annotated using Prokka v1.5 [17] and a genus-specific database [18]. The PacBio sequencing reads covered the finished assembly to an average depth of 148.01 X. To check the accuracy

of the PacBio assembly, the corresponding Illumina short-reads were mapped to the assembly using SMALT v0.5.8 (http://www.sanger.ac.uk/science/tools/smalt-0), with a maximum and minimum insert size of 1000 and 50, respectively. No single nucleotide polymorphisms were identified in the assembly upon mapping of these data.

Plasmid extraction, PCR, and molecular cloning

Plasmid DNA was isolated from *Escherichia coli* cultures using the QIAprep Spin Miniprep kit (Qiagen, #27104). Reaction intermediates were purified using the QIAquick PCR Purification kit (Qiagen, #28104). To clone $bla_{CARB\text{-}like}$, primers oMJD96 and oMJD97 were used to amplify $bla_{CARB\text{-}like}$ from MJD382 gDNA. The primers were designed to incorporate restriction enzyme sites and STOP codons as outlined in Figure S7. PCR was carried out using Phusion® high-fidelity DNA polymerase (NEB, # M0530S) according to the manufacturer's instructions, and a 10 mM dNTP mix (Thermo Scientific, #R0191). Twenty-nine PCR cycles were performed (annealing temperature: 65 ˚C; extension time: 90 sec). Amplicons were purified, and both this insert and the vector plasmid were digested using 20 units each of BamHI and SalI (NEB, #R3136S and #R3138S respectively) in CutSmart buffer for 15 min at 37 ˚C. The pACYC184 digest was then supplemented with 1 unit of rSAP (NEB, #M0371S), and both digestion reactions were continued for 30 min before digested DNA was purified. Digested insert and vector were mixed in a molar ratio of approximately 3:1, and ligated using T4 DNA ligase (NEB, #M0202S) at room temperature for 150 min. A ligase-minus reaction was included as a negative control. Ligase activity was inhibited by heating the mixtures to 65 ˚C for 10 min.

Five microlitres of ligation mixtures were used to transform competent *E. coli* (NEB, #C2987I) according to the manufacturer's instructions. Transformants were selected for on

solid LB media. *E. coli* that exhibited resistance to both ampicillin and chloramphenicol upon transformation were cultured and stored as glycerol stocks; these were also confirmed to be tetracycline-sensitive. Plasmids were prepared from these transformants as described above. The presence of $bla_{CARB\text{-}like}$ in pMJD61 was checked by PCR using oMJD88 and oMJD89 (homologous to $bla_{CARB\text{-}like}$), and confirmed by Sanger sequencing (GATC/Eurofins) with oMJD98 and oMJD99 (homologous to the sequences flanking *tet* on pACYC184).

Growth curves

Single colonies of *V. cholerae* were suspended in 0.5 ml LB broth by vortexing (10 sec). Two microlitres of this suspension were used to inoculate 150 µl LB in a 96-well microtitre plate (Corning CoStar #3595, flat-bottomed). A gas-permeable seal was applied to the plate, which was incubated at 37 ˚C with shaking in a BMG Fluostar Omega microtitre plate reader for 24 h. Absorbance (λ = 600 nm) was measured *per* well every 15 min (cycle time: 900 sec); four readings *per* well *per* timepoint were taken in a square matrix and averaged, to minimise the risk that debris or aggregated cells passing through the light beam would artificially increase absorbance readings. The plate was shaken at 300 rpm using the ‘meander corner well shaking’ mode. Three colonies *per* strain (biological triplicate) were used to inoculate six technical replicate cultures each. Raw growth curve data are available to download from the Figshare repository supporting this study.

**Accessory virulence gene**	**Locus ID in N16961 reference genome**	**Present in NCTC 30 (Percentage identity of translated protein)**
Zona occludens toxin (Zot)	*VC_1458*	No (CTXφ)
Accessory cholera enterotoxin (Ace)	*VC_1459*	No (CTXφ)
Haemolysin (*hlyA*)	*VC_A0219*	Yes (98%)
Mannose-sensitive haemagglutinin (MSHA)	*VC_0398..VC_0414*	Yes, see Figure S6
MARTX toxin (*rtxA*)	*VC_1451*	Yes (93%)
MARTX toxin accessory gene (*rtxC*)	*VC_1450*	Yes (100%)
HA/protease (*hapA*)	*VC_A0865*	Yes (98%)
Integrative conjugative element SXT/R391	Absent (if present, integrates into *VC_0659*)	No, *VC_0659* homologue is intact
Heat-stable enterotoxin NAG-ST (Genbank accession M85198.1)	Absent	No

Table S3. Presence of select accessory virulence factor genes in the NCTC 30 genome.

Identity percentages were calculated by alignment of protein sequences from NCTC 30 and N16961 using BLASTp. The presence of genes encoding MSHA is also illustrated in Figure S6. Since N16961 does not harbour NAG-ST, the translated NAG-ST nucleotide sequence (accession M85198.1) was used as a tBLASTx query to scan the NCTC 30 genome assembly for the presence of this enterotoxin.

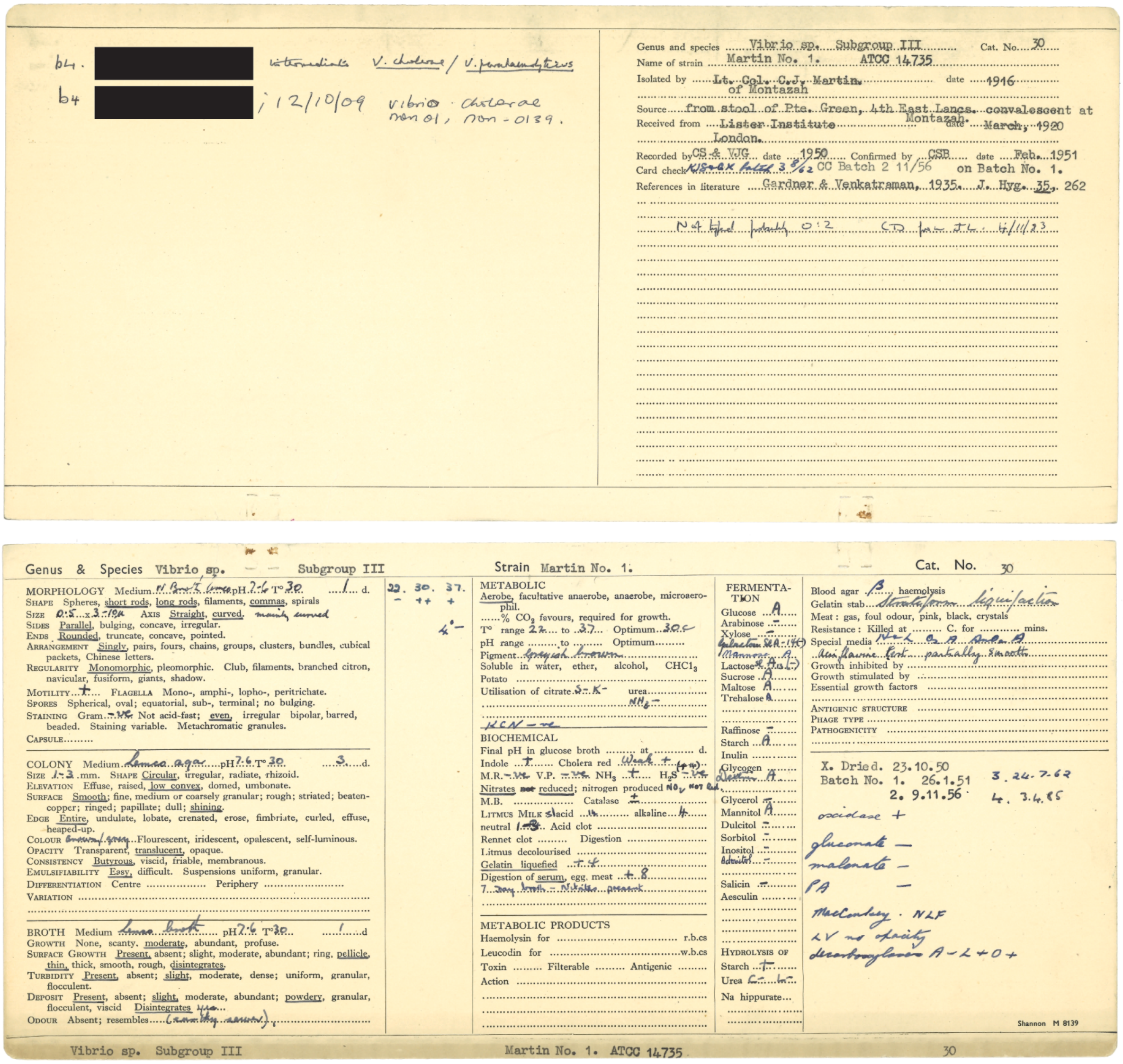
b4. [redacted] [illegible]
b4 [redacted] ; 12/10/09 vibrio cholerae non 01, non-0139.

Genus and species Vibrio sp. Subgroup III Cat. No. 30
Name of strain Martin No. 1. ATCC 14735
Isolated by Lt. Col. C.J. Martin. of Montazah date 1916
Source from stool of Pte. Green, 4th East Lancs. convalescent at Montazah.
Received from Lister Institute London. date March, 1920
Recorded by CS & VJG date 1950 Confirmed by CSB date Feb. 1951
Card check KJS + GX batch 3 8/62 CC Batch 2 11/56 on Batch No. 1.
References in literature Gardner & Venkatraman, 1935. J. Hyg. 35, 262

Genus & Species Vibrio sp. Subgroup III Strain Martin No. 1. Cat. No. 30

MORPHOLOGY Medium [illegible] pH 7·6 T° 30 1 d.
SHAPE Spheres, short rods, long rods, filaments, commas, spirals
SIZE 0·5 x 3-10µ AXIS Straight, curved. mainly curved
SIDES Parallel, bulging, concave, irregular.
ENDS Rounded, truncate, concave, pointed.
ARRANGEMENT Singly, pairs, fours, chains, groups, clusters, bundles, cubical packets, Chinese letters.
REGULARITY Monomorphic, pleomorphic. Club, filaments, branched citron, navicular, fusiform, giants, shadow.
MOTILITY + FLAGELLA Mono-, amphi-, lopho-, peritrichate.
SPORES Spherical, oval; equatorial, sub-, terminal; no bulging.
STAINING Gram -ve Not acid-fast; even, irregular bipolar, barred, beaded. Staining variable. Metachromatic granules.
CAPSULE

22. 30. 37.
− ++ +
4° −

COLONY Medium lemco agar pH 7·6 T° 30 3 d.
SIZE 1-3 mm. SHAPE Circular, irregular, radiate, rhizoid.
ELEVATION Effuse, raised, low convex, domed, umbonate.
SURFACE Smooth; fine, medium or coarsely granular; rough; striated; beaten-copper; ringed; papillate; dull; shining.
EDGE Entire, undulate, lobate, crenated, erose, fimbriate, curled, effuse, heaped-up.
COLOUR brownish/grey Flourescent, iridescent, opalescent, self-luminous.
OPACITY Transparent, translucent, opaque.
CONSISTENCY Butyrous, viscid, friable, membranous.
EMULSIFIABILITY Easy, difficult. Suspensions uniform, granular.
DIFFERENTIATION Centre Periphery
VARIATION

BROTH Medium lemco broth pH 7·6 T° 30 1 d
GROWTH None, scanty. moderate, abundant, profuse.
SURFACE GROWTH Present, absent; slight, moderate, abundant; ring, pellicle, thin, thick, smooth, rough, disintegrates.
TURBIDITY Present, absent; slight, moderate, dense; uniform, granular, flocculent.
DEPOSIT Present, absent; slight, moderate, abundant; powdery, granular, flocculent, viscid Disintegrates yes
ODOUR Absent; resembles (earthy, [illegible])

METABOLIC
Aerobe, facultative anaerobe, anaerobe, microaerophil.
% CO_2 favours, required for growth.
T° range 22 to 37 Optimum 30°C
pH range to Optimum
Pigment greyish brown
Soluble in water, ether, alcohol, $CHCl_3$
Potato
Utilisation of citrate S− K− urea
NH_3 −
KCN −ve

BIOCHEMICAL
Final pH in glucose broth at d.
Indole + Cholera red Weak + (+ 14)
M.R. −ve V.P. −ve NH_3 + H_2S −ve
Nitrates not reduced; nitrogen produced NO₂ not [illegible]
M.B. Catalase +
LITMUS MILK sl acid 14 alkaline 4
neutral 1-3 Acid clot
Rennet clot Digestion
Litmus decolourised
Gelatin liquefied + 4
Digestion of serum, egg, meat + 8
7 day broth − Nitrites present

METABOLIC PRODUCTS
Haemolysin for r.b.cs
Leucodin for w.b.cs
Toxin Filterable Antigenic
Action

FERMENTATION
Glucose A
Arabinose −
Xylose −
Galactose [illegible]
Mannose A
Lactose sl. A [illegible] (−)
Sucrose A
Maltose A
Trehalose A
Raffinose −
Starch A
Inulin
Glycogen
Dextrin A
Glycerol −
Mannitol A
Dulcitol −
Sorbitol −
Inositol −
Adonitol −
Salicin −
Aesculin
HYDROLYSIS OF
Starch +
Urea [illegible]
Na hippurate

Blood agar β haemolysis
Gelatin stab stratiform liquifaction
Meat: gas, foul odour, pink, black, crystals
Resistance: Killed at C. for mins.
Special media [illegible]
acriflavine test partially smooth
Growth inhibited by
Growth stimulated by
Essential growth factors
ANTIGENIC STRUCTURE
PHAGE TYPE
PATHOGENICITY

X. Dried. 23.10.50
Batch No. 1. 26.1.51
2. 9.11.56
3. 24-7-62
4. 3.4.85
oxidase +
gluconate −
malonate −
PA −
MacConkey · NLF
LV no opacity
decarboxylases A − L + O +

Shannon M 8139

Vibrio sp. Subgroup III Martin No. 1. ATCC 14735 30

Figure S1. The NCTC quality check card for NCTC 30. This isolate has been passaged four times since its initial lyophilisation in 1950. Batch 3, prepared in 1962, was investigated and sequenced in this study. Information which is not relevant to the conclusions drawn in this study has been redacted (black rectangles).

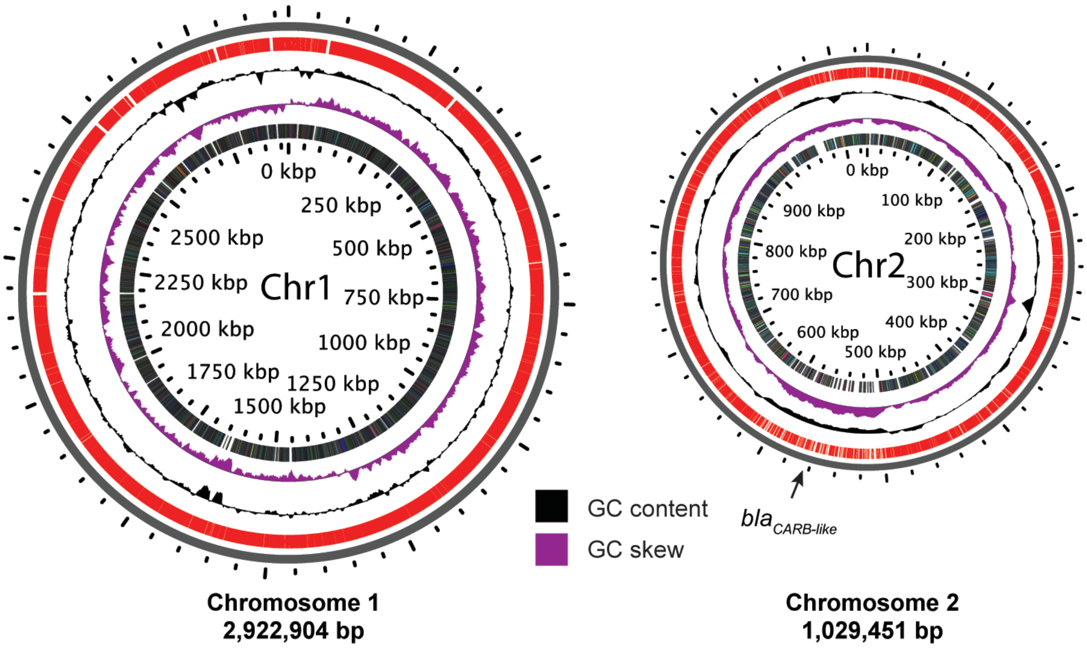

Figure S2. Visualisation of the NCTC 30 genome assembly. Single contigs were assembled for both of the chromosomes of NCTC 30. A β-lactamase gene was identified within the AT-rich super-integron of chromosome 2.

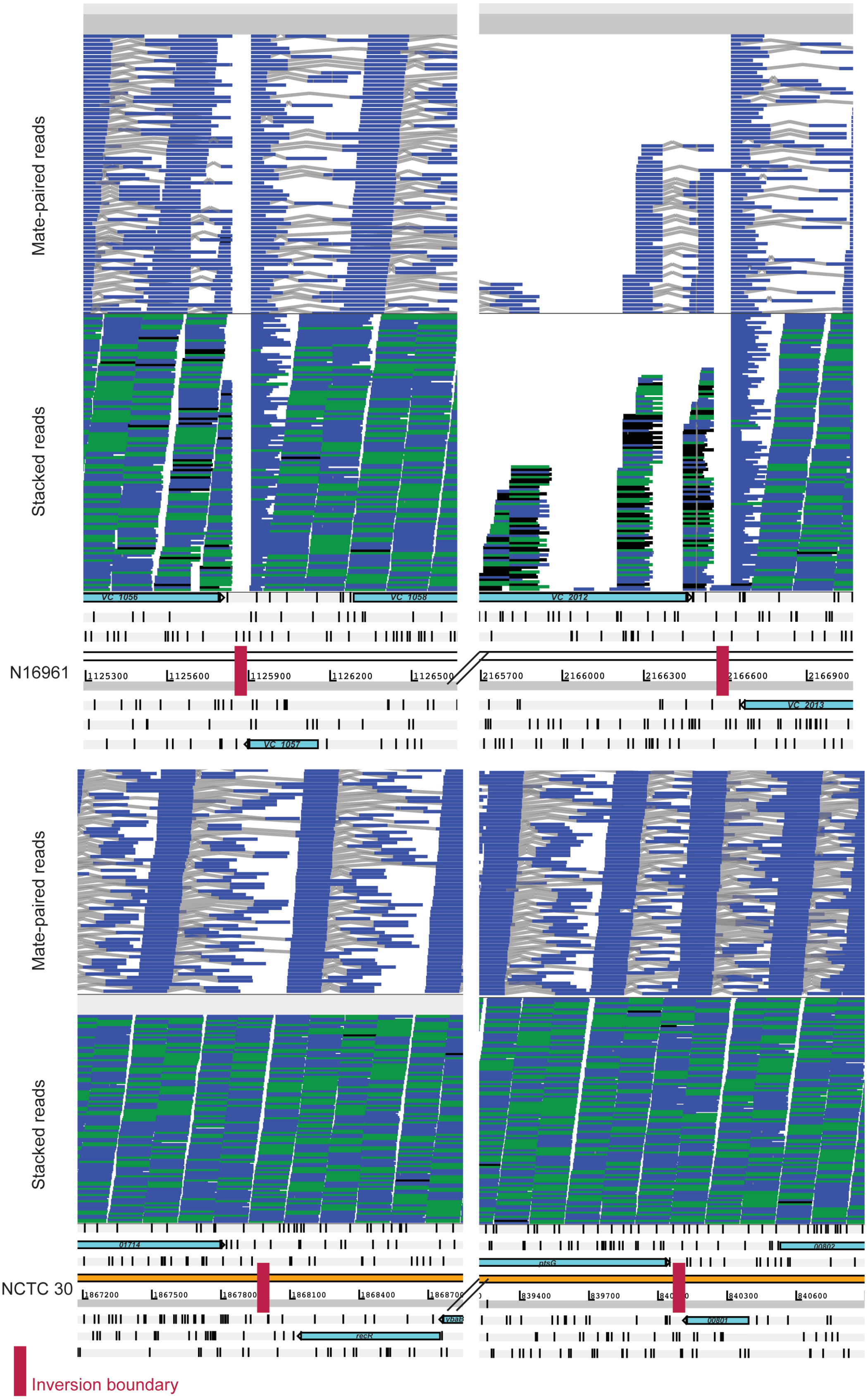

Figure S3. Confirming the NCTC 30 chromosome 1 rearrangement using mate-paired Illumina reads. Illumina reads for NCTC 30 were mapped to the N16961 and NCTC 30

genome sequences, and the mapped reads were visualised using Artemis and BamView [35,36]. The regions that delineate the genomic inversion shown in Figure 1B are presented. In N16961, no reads map to the inversion region adjacent to *VC_1057*, and none of the reads that map to one of adjacent sequences have a mate-pair on the other side of the inversion site. A similar observation was made at the inversion site at *VC_2013*. However, the corresponding inversion sites in NCTC 30 have reads that map to the inversion site sequence, as well as reads mapping to one side which have mate-paired reads on the other side of the inversion site. The gene *VC_2012* is poorly conserved between NCTC 30 and N16961; hence, there are few NCTC 30 reads that map to this region. Hatch marks denote a truncation of the genome sequence view.

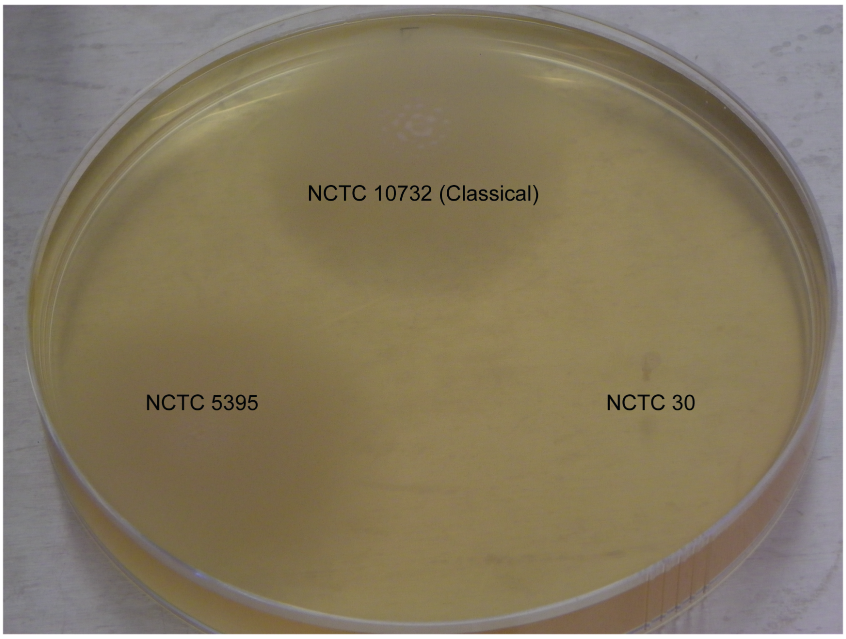

LB supplemented with 0.3% agar

Figure S4. NCTC 30 displays a non-motile phenotype. Soft agar plates were incubated for 18 hours before imaging.

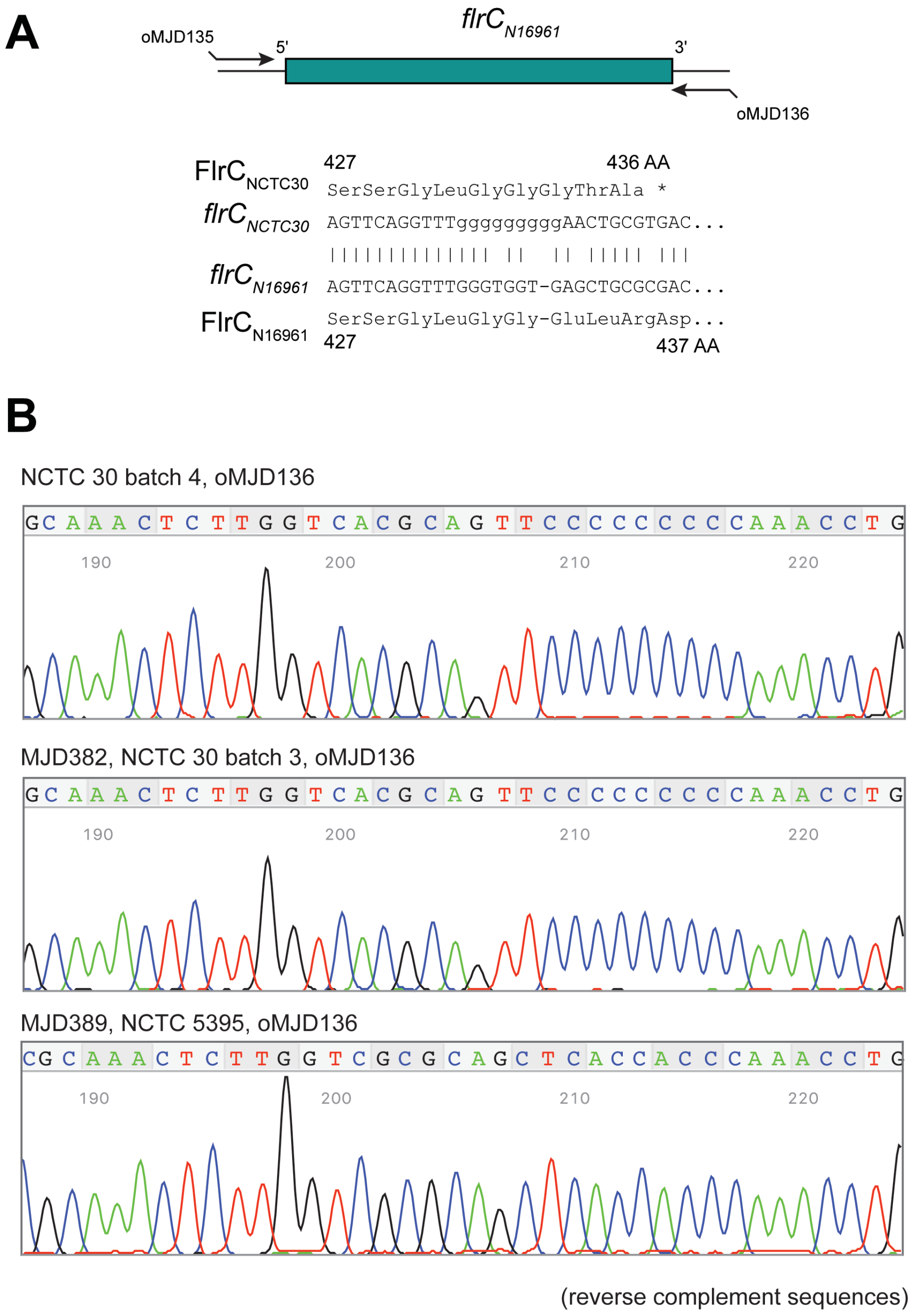

Figure S5. Confirming *flrC* mutations by amplicon sequencing. (A): A schematic of *flrC* in the N16961 genome is reported. The binding sites and orientation of primers oMJD135 and oMJD136 are indicated, as are the location and sequence of the *flrC* frameshift in NCTC 30. (B): Amplicon sequencing shows that the nine G-C base pairs in NCTC 30 batch 3 are also present in the batch 4 DNA preparation. Sequencing traces were visualised using 4Peaks (https://nucleobytes.com/4peaks/index.html). Raw sequencing files are available in Figshare.

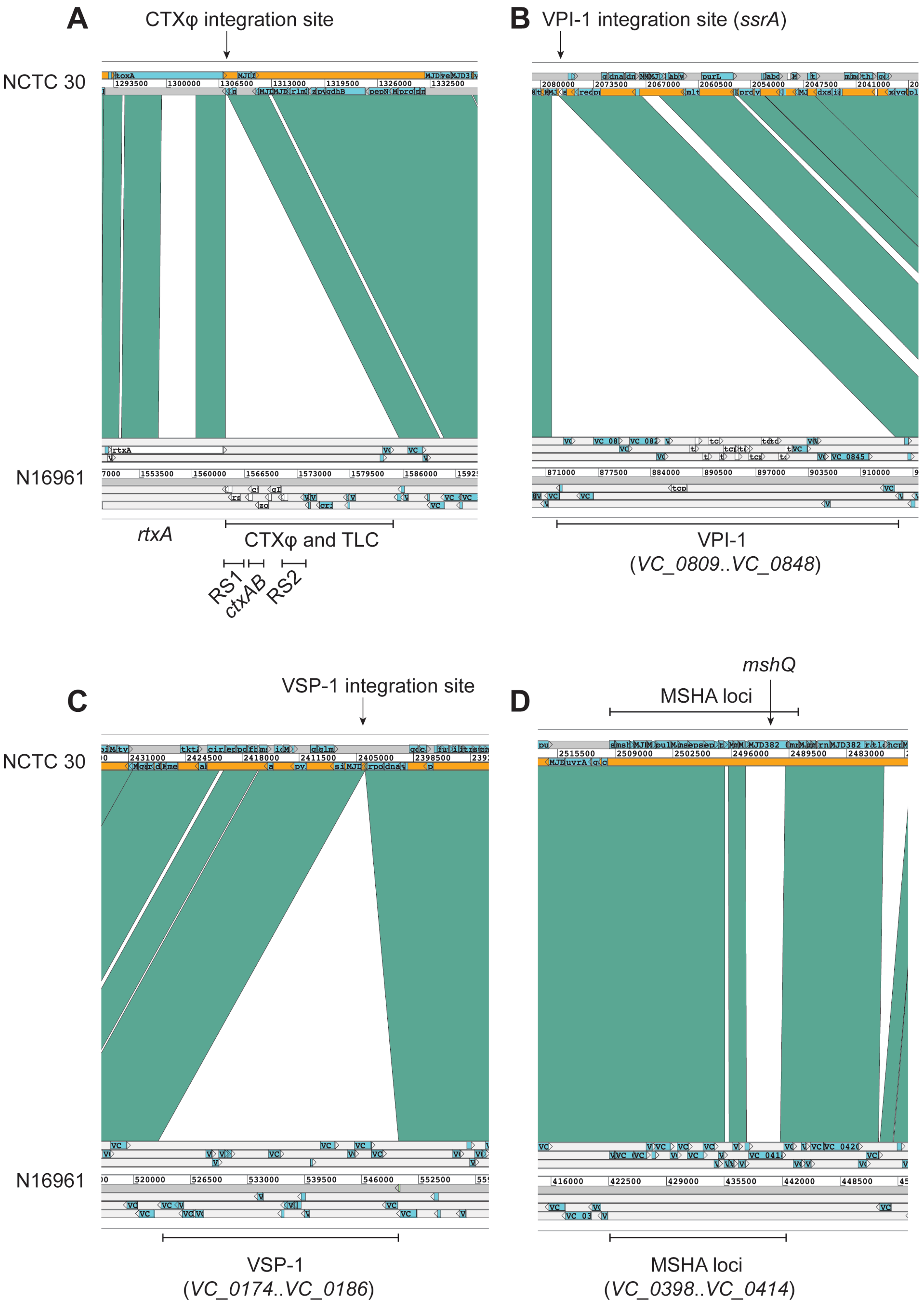

Figure S6. Presence and absence of virulence-associated genes in NCTC 30. Synteny plots were produced using ACT [23] to visualise the presence and absence of nucleotide

sequences in the NCTC 30 genome assembly. When compared to the N16961 reference sequence, the absence of CTXφ (A), VPI-1 (B) and VSP-1 (C) from NCTC 30 is evident. No sequences homologous to these elements were detected anywhere in the NCTC 30 assembly. The genes that encode the MSHA accessory virulence determinant are present in NCTC 30 (D), although the NCTC 30 *mshQ* gene is dissimilar to that of N16961.

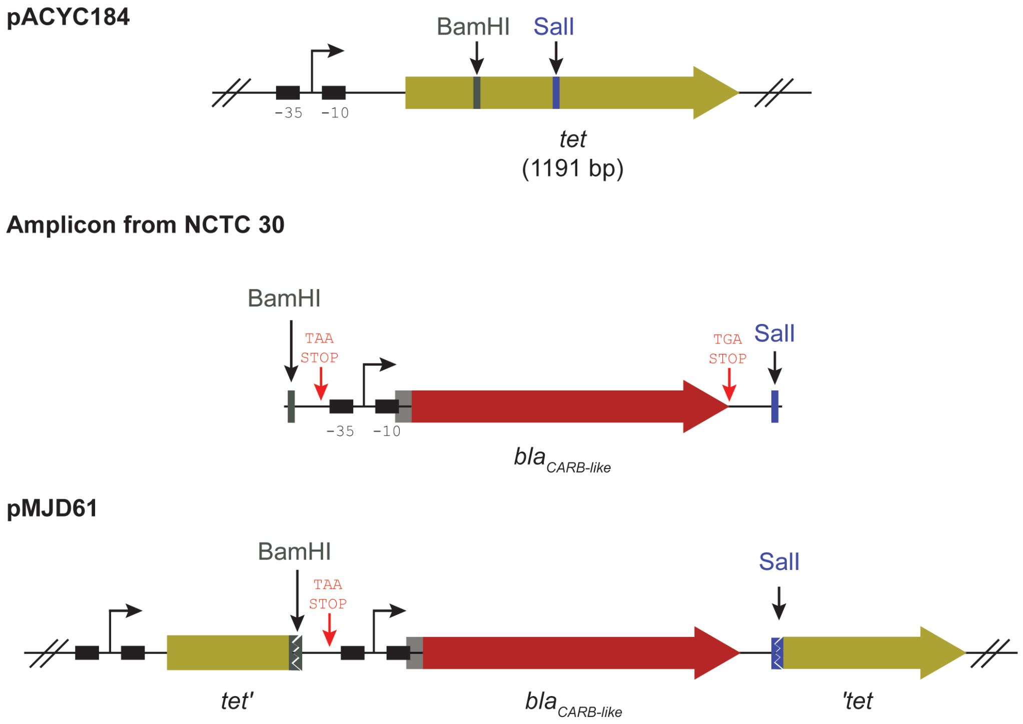

Figure S7. Strategy for cloning *bla*$_{CARB\text{-}like}$ into a low-copy plasmid. pACYC184 is a low-copy cloning vector that encodes resistance to both chloramphenicol and tetracycline. *bla*$_{CARB\text{-}like}$ was amplified from the NCTC 30 genome using primers oMJD96 and oMJD97, incorporating BamHI and SalI restriction sites. The amplicon was inserted into the pACYC184 *tet* gene, such that a premature STOP codon would be introduced in-frame into *tet*. An analysis of the sequence of *bla*$_{CARB\text{-}like}$ using BPROM (http://softberry.com) predicted *E. coli* σ^{70} -35 and -10 elements within the insert (indicated). Although this prediction identifies *E. coli* promoter elements, not those from *V. cholerae*, these elements might provide a native promoter from which to drive *bla*$_{CARB\text{-}like}$ expression in *E. coli*. Figures are not to scale.

Supplementary references for genomes used in this study.

1. Haley BJ *et al.* 2010 Comparative genomic analysis reveals evidence of two novel *Vibrio* species closely related to *V. cholerae*. *BMC Microbiol.* **10**, 154. (doi:10.1186/1471-2180-10-154)

2. Orata FD, Kirchberger PC, Méheust R, Barlow EJ, Tarr CL, Boucher Y. 2015 The dynamics of genetic interactions between *Vibrio metoecus* and *Vibrio cholerae*, two close relatives co-occurring in the environment. *Genome Biol. Evol.* **7**, 2941–2954. (doi:10.1093/gbe/evv193)

3. Watve SS, Chande AT, Rishishwar L, Mariño-Ramírez L, Jordan IK, Hammer BK. 2016 Whole-genome sequences of 26 *Vibrio cholerae* isolates. *Genome Announc.* **4**, e01396-16. (doi:10.1128/genomeA.01396-16)

4. Hasan NA *et al.* 2012 Genomic diversity of 2010 Haitian cholera outbreak strains. *Proc. Natl. Acad. Sci.* **109**, E2010–E2017. (doi:10.1073/pnas.1207359109)

5. Chowdhury F *et al.* 2015 *Vibrio cholerae* serogroup O139: Isolation from cholera patients and asymptomatic household family members in Bangladesh between 2013 and 2014. *PLoS Negl. Trop. Dis.* **9**, e0004183. (doi:10.1371/journal.pntd.0004183)

6. Domman D *et al.* 2017 Integrated view of *Vibrio cholerae* in the Americas. *Science* **358**, 789–793. (doi:10.1126/science.aao2136)

7. Chun J *et al.* 2009 Comparative genomics reveals mechanism for short-term and long-term clonal transitions in pandemic *Vibrio cholerae*. *Proc. Natl. Acad. Sci.* **106**, 15442–15447. (doi:10.1073/pnas.0907787106)

8. Haley BJ *et al.* 2014 Genomic and phenotypic characterization of *Vibrio cholerae* non-O1 isolates from a US Gulf Coast cholera outbreak. *PLoS ONE* **9**, e86264. (doi:10.1371/journal.pone.0086264)

9. Johnson SL *et al*. 2015 Complete genome assemblies for two single-chromosome *Vibrio cholerae* isolates, strains 1154-74 (serogroup O49) and 10432-62 (serogroup O27). *Genome Announc*. **3**. (doi:10.1128/genomeA.00462-15)

10. Choi SY *et al.* 2016 Phylogenetic diversity of *Vibrio cholerae* associated with endemic cholera in Mexico from 1991 to 2008. *mBio* **7**, e02160-15. (doi:10.1128/mBio.02160-15)

11. Thompson CC, Marin MA, Dias GM, Dutilh BE, Edwards RA, Iida T, Thompson FL, Vicente ACP. 2011 Genome sequence of the human pathogen *Vibrio cholerae* Amazonia. *J. Bacteriol.* **193**, 5877–5878. (doi:10.1128/JB.05643-11)

12. Islam A *et al.* 2013 Indigenous *Vibrio cholerae* strains from a non-endemic region are pathogenic. Open Biol. **3**, 120181. (doi:10.1098/rsob.120181)

13. Kiiru J *et al*. 2013 A study on the geophylogeny of clinical and environmental *Vibrio cholerae* in Kenya. *PLoS ONE* **8**, e74829. (doi:10.1371/journal.pone.0074829)

14. Pérez Chaparro PJ *et al*. 2011 Whole genome sequencing of environmental *Vibrio cholerae* O1 from 10 nanograms of DNA using short reads. *J. Microbiol. Methods* **87**, 208–212. (doi:10.1016/j.mimet.2011.08.003)

15. Azarian T *et al*. 2016 Non-toxigenic environmental *Vibrio cholerae* O1 strain from Haiti provides evidence of pre-pandemic cholera in Hispaniola. *Sci. Rep.* **6**, srep36115. (doi:10.1038/srep36115)

16. Feng L *et al.* 2009 A recalibrated molecular clock and independent origins for the cholera pandemic clones. *PLoS ONE* **3**, e4053. (doi:10.1371/journal.pone.0004053)

17. Mutreja A *et al.* 2011 Evidence for several waves of global transmission in the seventh cholera pandemic. *Nature* **477**, 462–465. (doi:10.1038/nature10392)

18. Hu D, Liu B, Feng L, Ding P, Guo X, Wang M, Cao B, Reeves PR, Wang L. 2016 Origins of the current seventh cholera pandemic. *Proc. Natl. Acad. Sci.* **113**, E7730–E7739. (doi:10.1073/pnas.1608732113)

19. Allué-Guardia A, Echazarreta M, Koenig SSK, Klose KE, Eppinger M. 2018 Closed genome sequence of *Vibrio cholerae* O1 El Tor Inaba strain A1552. *Genome Announc.* **6**, e00098-18. (doi:10.1128/genomeA.00098-18)

20. Morais LLC de S *et al*. 2012 Complete genome sequence of a sucrose-nonfermenting epidemic strain of *Vibrio cholerae* O1 from Brazil. *J. Bacteriol.* **194**, 2772–2772. (doi:10.1128/JB.00300-12)

21. Heidelberg JF *et al*. 2000 DNA sequence of both chromosomes of the cholera pathogen *Vibrio cholerae*. *Nature* **406**, 477–483. (doi:10.1038/35020000)

22. Grim CJ *et al*. 2010 Genome sequence of hybrid *Vibrio cholerae* O1 MJ-1236, B-33, and CIRS101 and comparative genomics with *V. cholerae*. *J. Bacteriol.* **192**, 3524–3533. (doi:10.1128/JB.00040-10)

23. Didelot X, Pang B, Zhou Z, McCann A, Ni P, Li D, Achtman M, Kan B. 2015 The role of China in the global spread of the current cholera pandemic. *PLoS Genet.* **11**, e1005072. (doi:10.1371/journal.pgen.1005072)

24. Reimer AR *et al*. 2011 Comparative genomics of *Vibrio cholerae* from Haiti, Asia, and Africa. *Emerg. Infect. Dis.* **17**, 2113–2121. (doi:10.3201/eid1711.110794)

25. Folster JP, Katz L, McCullough A, Parsons MB, Knipe K, Sammons SA, Boncy J, Tarr CL, Whichard JM. 2014 Multidrug-resistant IncA/C plasmid in *Vibrio cholerae* from Haiti. *Emerg. Infect. Dis.* **20**, 1951–1952. (doi:10.3201/eid2011.140889)

26. Dillon MM, Sung W, Sebra R, Lynch M, Cooper VS. 2017 Genome-wide biases in the rate and molecular spectrum of spontaneous mutations in *Vibrio cholerae* and *Vibrio fischeri*. *Mol. Biol. Evol.* **34**, 93–109. (doi:10.1093/molbev/msw224)

27. Okada K, Na-Ubol M, Natakuathung W, Roobthaisong A, Maruyama F, Nakagawa I, Chantaroj S, Hamada S. 2014 Comparative genomic characterization of a Thailand-Myanmar isolate, MS6, of *Vibrio cholerae* O1 El Tor, which is phylogenetically related to a 'US Gulf Coast' clone. *PLoS ONE* **9**, e98120. (doi:10.1371/journal.pone.0098120)
